# Is Social Media Influencing Patients to Choose Aligner Treatment?

**DOI:** 10.7759/cureus.86523

**Published:** 2025-06-22

**Authors:** Amol S Patil, Advait R Kshirsagar, Saaransh Handa

**Affiliations:** 1 Orthodontics and Dentofacial Orthopaedics, Bharati Vidyapeeth (Deemed to be University) Dental College and Hospital, Pune, Pune, IND

**Keywords:** advertising, aligners, customer, orthodontic treatment, social media

## Abstract

Objective

This study aimed to determine the influence of social media advertising on patients’ decision to choose aligner treatment.

Participants

Three hundred valid responses were collected from patients across genders and age groups who desire aligner treatment/undergoing aligner treatment/completed aligner treatment directly or through their orthodontists/dental surgeons.

Materials and methods

A questionnaire-style survey was created to record the form of media advertising so as to record the data obtained in a format suitable for analysis at Bharati Vidyapeeth (Deemed to be University) Dental College and Hospital, Pune. The questionnaire was shared with orthodontists and general dentists who forwarded it to their patients who are undergoing or have completed aligner treatment and also to patients interested in or undergoing DIY (do it yourself) aligner treatment via social media groups and messaging platforms.

Results

Among 300 respondents, 94.6% reported a moderate to very high influence of social media and 63.6% reported high to very high influence. Gender-based analysis showed significantly higher influence among females (62.9%) as compared to males (37.1%). Respondents aged 20-40 years were most influenced by social media. Digital advertisements were significantly preferred across age groups; men favored word of mouth, while women preferred mixed strategies. Instagram (68.6%) was the most influential platform, followed by YouTube (56.2%). Perceived usefulness of social media also showed a significant gender difference (χ² = 19.931, p = 0.018).

Conclusions

Social media significantly influences aligner-related decision-making, especially among women and individuals aged 20-40. Instagram and YouTube are dominant platforms. Dental Practitioners should harness social media responsibly to improve patient education and counter misinformation.

## Introduction

In contemporary societies, like any other professional service, the majority of dental healthcare professionals compete for consumers. Marketing is crucial in the retail sector to showcase products or services in a way that increases their appeal to potential customers [1]. This is frequently used in orthodontics, a discipline that occasionally has a reputation for providing permissive services. Aligners have gained immense popularity among the youth during the past 10 years since conventional orthodontic treatment compromises the appearance of an individual during treatment [2,3].

The wide traction of aligners has led to the introduction of DIY (do it yourself) aligner treatment, a clear appliance system that does not need an interaction between the patient and the dental operator [4]. The main benefit of clear aligner treatment is improved aesthetics with a good patient acceptance rate and an overall higher standard of living [5]. The information about aligners on the internet has increased and more people use the Internet to gain knowledge about the various features of orthodontic treatment every day [6].

Orthodontic services taken by consumers are based on their attitude and other environmental factors. Advertisements play an important role in stimulating interest in such consumers based on their interests by educating them and having the ability to differentiate one practitioner from the others. However, due to economic considerations, ethical guidelines set forth by councils, and the belief that some consumers may elucidate advertising as a measure of lower quality of treatment many orthodontists are reluctant towards the use of media advertising [7].

Although some studies have evaluated the impact of the media on the choice of dental treatment for patients, there is a lack of research focusing specifically on how social media affect the decision to choose a coordinated treatment. This questionnaire-based study aims to understand the role of the media as a determining factor for the choice of aligner treatment. The purpose of this study is to provide insight into the sources of information and media influence on the selection of aligners by patients as a treatment option.

## Materials and methods

Questionnaire design and distribution

A questionnaire (https://forms.gle/HAvBgfoxxWv8PD9m7) (see Appendix) with eight questions was developed. The sample size for this study was 300 valid responses, and the sampling procedure used was convenience sampling, which was chosen due to ease of accessibility to patients undergoing or having undergone aligner treatment and the time-efficient nature of gathering data through clinicians and online platforms. While convenience sampling enabled rapid and targeted data collection from relevant respondents, we acknowledge that this method may introduce selection bias, as participants with greater digital literacy or social media use may be overrepresented. Response bias is also possible, as those with stronger opinions (positive or negative) may be more likely to respond. To mitigate this, responses were collected anonymously, and participants were informed of the study’s voluntary nature.

This structured, anonymous Google Forms questionnaire (Google LLC, Mountain View, California, United States) was distributed using a combination of clinician-led and social-media-based strategies: 1) Clinician referral: Orthodontists and general dentists were requested to circulate the survey among their aligner patients-both ongoing and completed cases-through clinic visits and follow-up communication. 2) Patient networks: The questionnaire was shared directly in DIY aligner interest groups via WhatsApp and Facebook Messenger, ensuring inclusion of patients not directly under clinical supervision. 3) Snowball sampling: Participants were encouraged to forward the form to peers with similar aligner treatment experience or interest.

Responses were collected from patients above 18 years of either gender who desire aligner treatment/undergoing aligner treatment/completed aligner treatment directly or through their orthodontists/dental surgeons at Bharati Vidyapeeth (Deemed to be University) Dental College and Hospital, Pune. While the primary inclusion criteria were participants above 18 years of age, a few responses were also collected from individuals aged 12-18 years who were undergoing or had undergone aligner treatment. For these participants, informed consent was obtained from their parents or legal guardians prior to participation in the study. All participants filled the questionnaire designed to examine their access to social media and their awareness and utilization of the same. Furthermore, it explored their willingness to engage in different forms of media to aid in their aligner treatment. The questionnaire provided demographic data, factors influencing selection of aligners and the utilization of different forms of media by the patients.

Questionnaire validation and pilot testing

Prior to full-scale deployment, the questionnaire underwent formal content validation by a panel of seven domain experts in orthodontics, public health, and survey methodology. Each item was rated independently for importance, relevance, and clarity using a four-point Likert scale (1 = not at all, 4 = highly). Based on these ratings, three indices were calculated: 1) Item-Level Content Validity Index (I-CVI): Proportion of experts rating an item 3 or 4. 2) Scale-Level Content Validity Index (S-CVI): Mean of I-CVIs across all items per domain. 3) Content Validity Ratio (CVR): Based on Lawshe’s method, quantifying the essentiality of each item.

All items achieved I-CVI values ≥ 0.86 in at least two domains, and CVR values ranged from 0.43 to 1.00, indicating strong agreement among experts as shown in Table 1. This confirms that the questionnaire items were conceptually relevant, clearly worded, and essential for capturing the intended constructs.

**Table 1:** Content validity indices and ratios for each item in the questionnaire

Q. No.	Questionnaire item	I-CVI (Importance)	I-CVI (Relevance)	I-CVI (Clarity)	CVR (Importance)	CVR (Relevance)	CVR (Clarity)
1	What is your age?	0.86	0.86	0.86	0.71	0.71	0.71
2	Gender	0.86	0.86	0.86	0.71	0.71	0.71
3	From where did you hear about aligners?	1.00	1.00	0.86	1.00	1.00	0.71
4	To what degree was the influence of media advertisements in you undergoing aligner treatment?	0.86	0.86	0.86	0.71	0.71	0.71
5	If you have used social media for information on aligners, did you find it useful?	1.00	1.00	1.00	1.00	1.00	1.00
6	What type of advertisement strategy do you prefer?	0.86	0.86	1.00	0.71	0.71	1.00
7	Do you think it’s important to scroll through relevant social media before seeking treatment?	0.86	0.86	0.71	0.71	0.71	0.43
8	Do you feel that social media made you better informed about aligners?	1.00	1.00	0.86	1.00	1.00	0.71

The S-CVI/Average (S-CVI/Ave) values for importance and relevance were greater than 0.90, indicating excellent content validity, whereas for clarity the value was 0.8638, which was considered acceptable. The S-CVI/Universal Agreement (S-CVI/UA) values were low by the virtue of it being a stricter index. The values for each criterion are shown in Table 2.

**Table 2:** Values for State Content Validity Index/Average (S-CVI/Ave) and State Content Validity Index/Universal Agreement (S-CVI/UA)

Metric	Importance	Relevance	Clarity
S-CVI/Ave	0.9125	0.9125	0.8638
S-CVI/UA	0.375	0.375	0.25

The questionnaire was tested in a pilot test with 30 respondents to assess clarity, relevance, and technical reliability. On the basis of the pilot, small language adjustments were made to better understand. The internal coherence of the final instrument was measured with Cronbach's alpha of 0.81, which shows satisfactory reliability in all assessed categories.

Statistical analysis

The sample size was calculated using an online sample size calculator (Raosoft) to achieve a 95% confidence level with a margin of error set at 6%. Assuming a large population of potential social media users and aligner seekers, the estimated minimum sample size was 266. To account for incomplete or invalid responses, 300 completed and valid responses were collected.

For all statistical tests, p < 0.05 was considered to be statistically significant. Results were obtained and analyzed using descriptive statistics. A chi-square test was used to evaluate the association between gender and age with source of information on aligners, degree of influence of social media advertisements and preferred advertisement strategy. Data was subjected to statistical analysis using IBM SPSS Statistics for Windows, Version 21.0 (released 2018, IBM Corp., Armonk, NY).

The Institutional Ethics Committee of Bharati Vidyapeeth (Deemed to be University) Dental College and Hospital, Pune, issued approval EC/NEW/INST/2021/MH/0029.

## Results

A total of 300 individuals participated in the study, representing a wide range of age and gender groups. The sample consisted of 111 males and 189 females. The age distribution showed that most respondents (n = 184, 61.3%) were aged 20 to 40, followed by 59 individuals aged 12 to 19 (19.7%) and 57 individuals aged over 40 (19.0%) as shown in Table 3.

**Table 3:** Demographic distribution

Age group	Male	Female	Total
12–19	26	33	59
20–40	60	124	184
>40	25	32	57

The questionnaire demonstrated satisfactory internal consistency across all evaluated categories (Cronbach’s alpha = 0.81). Of the 300 respondents, 286 individuals (95.3%) reported being influenced by social media to undergo aligner treatment. Among these, 187 respondents (62.33%) cited a high to very high influence, while 89 respondents (29.66%) reported a moderate level of influence. Only 24 respondents (8%) indicated a low impact of social media on their decision to choose aligners.

A significant gender difference was observed, 81 out of 189 females (42.8%) and just 35 out of 111 males (31.5%) were highly influenced by social media advertisements to choose aligner treatment (χ² = 23.344, p = 0.025), as shown in Table 4.

**Table 4:** Chi-square test of degree of influence of social media advertisements in undergoing aligner treatment and gender

Question		Gender		
To what degree was the influence of media advertisements in you undergoing aligner treatment?	Response	Female	Male	Total
	High	81	35	116
	Low	13	11	24
	Medium	52	37	89
	Very High	43	28	71
Total		189	111	300

Furthermore, women perceived social media to be more useful for aligner-related decisions than men (χ² = 19.931, p = 0.018), as shown in Table 5. 

**Table 5:** Chi-square test of usefulness of social media for information on aligners and gender

Question	Response	Female	Male	Total
If you have used social media for information on aligners, did you find it useful?	Yes	54	29	83
	No	13	19	32
	Maybe	121	65	186
Total		189	111	300

**Figure 1:**
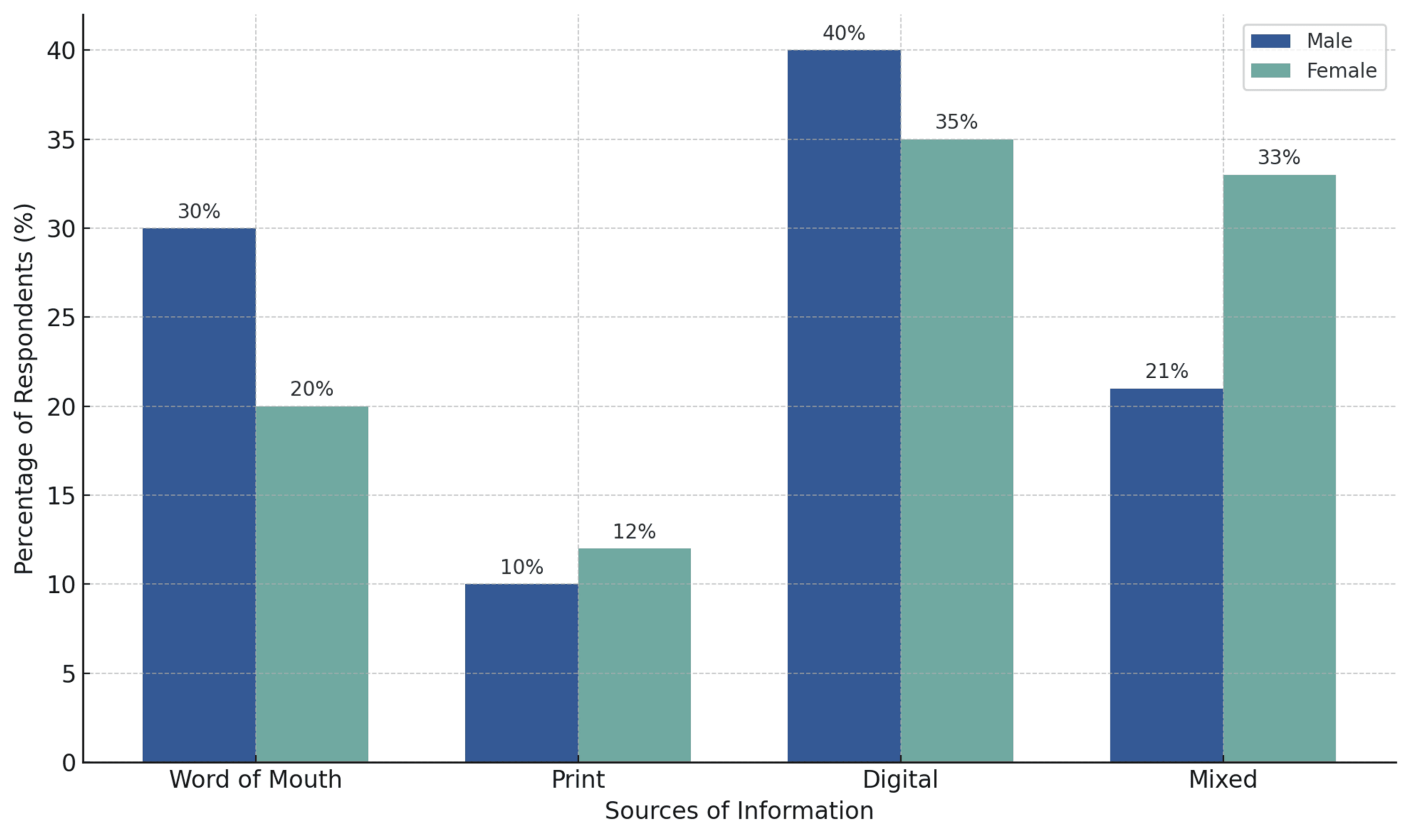
Advertisement preference by gender

As shown in Figure 1, digital advertisements emerged as the most influential source across genders, with 40% of males and 35% of females reporting digital media as their primary source of information. A notable gender difference was observed in the preference for word-of-mouth recommendations, with 30% of male participants favoring it compared to only 20% of females. On the contrary, a higher proportion of females (33%) preferred a mixed media approach comprising digital, print and interpersonal sources as compared to 21% of males. Print media was the least preferred across both groups, with only 10% of males and 12% of females citing it as their main source of information. 

**Figure 2:**
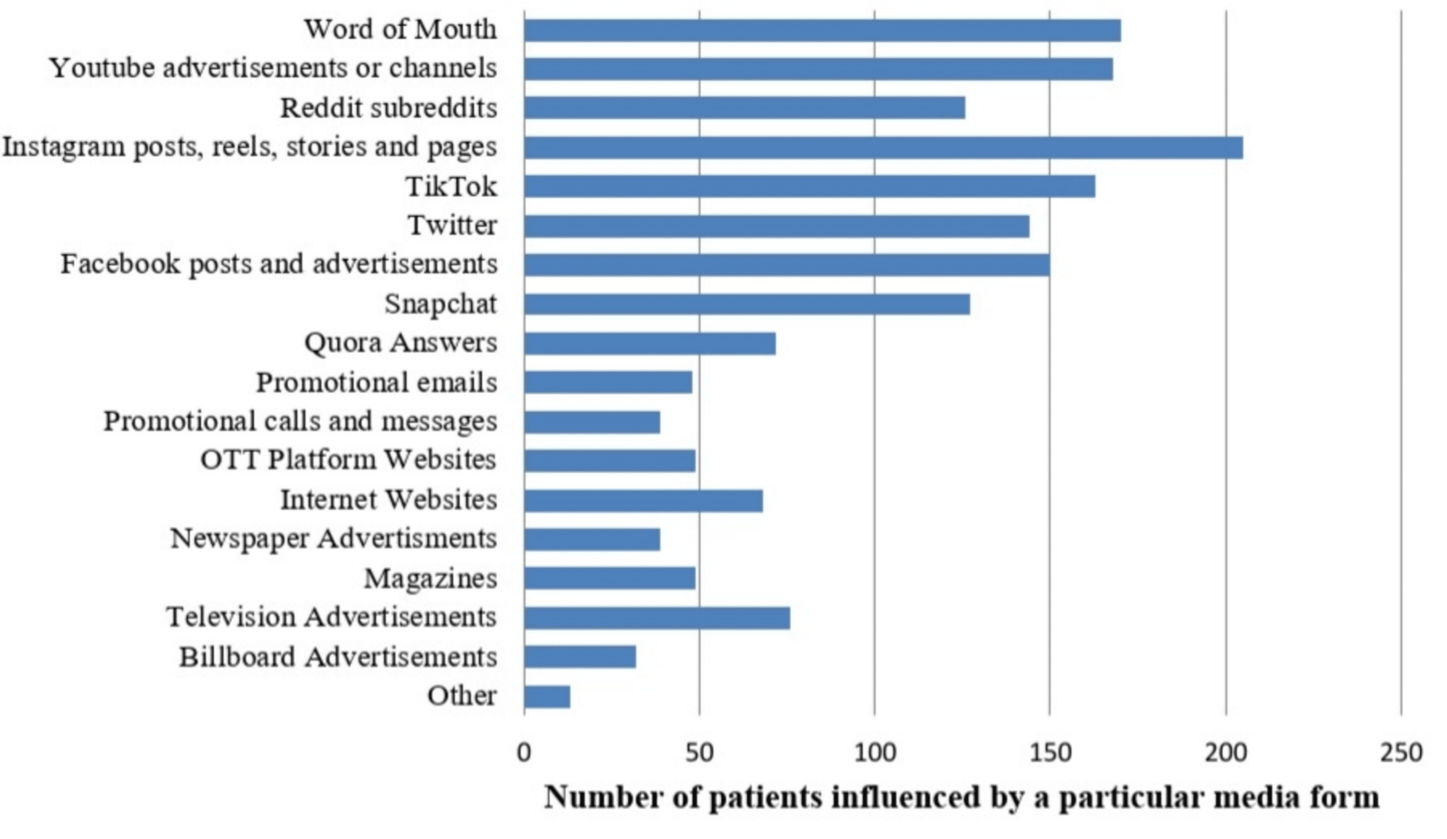
Proportion of various social media influencing patients decision to choose aligners

As shown in Figure 2, Instagram was reported as the most influential platform with 206 respondents (66.6%), followed by YouTube (66.5%), Facebook (151 respondents) and Twitter (44.2%). On the other hand, traditional media such as television, magazines and billboards have less influence. Among all genders and age groups, the majority of participants reported that reviewing social media content prior to treatment made them feel better informed and more confident in their choice of aligners. 

## Discussion

Social media for advertising is considered a form of inbound marketing, which focuses on providing help and information to buyers when they want help and is generally considered a highly favored factor in marketing.

According to our study, females cited a high influence of social media (62.9%) as compared to men (37.1%), and the results are consistent with the results of previous studies [8]. Between the ages of 20 and 40, a high influence of social media was cited, which is attributable to the increased use of smartphones and is consistent with the results of the study by Tammisalo et al. (2022) [9]. The advent of social media has proved to be capable to modify the way people perceive certain information. This is because they provide "grouped" users (such as Facebook and LinkedIn groups) based on their preferences. Being surrounded by people with very similar perceptions of reality, even de facto, can confuse objectivity and criticism. As a result, social media is deemed highly influential in the decision-making process [10]. This is in confluence with the results of our study, which show that most participants cited a high (39.5%) to very high (24.1%) effect of social media in their decision to undergo aligner treatment and most of them found it to be useful. Few respondents cited a low impact of social media on their decision to choose aligners.

Digital advertisement was the most preferred form of advertisement according to the respondents (85.9%). This is in agreement with the results of the study by Sha et al. (2017) that attributed it to digital media being more persuasive and less intrusive [11]. However, respondents above the age of 40 preferred a combined digital and print form of advertisement.

Instagram use among orthodontists and aligner providers has been increasing at a rapid rate in recent years. Our study showed that Instagram reels and pages were seen to be the source which influenced most patients (68.6% respondents). According to Saternus et al. (2022), this might be because viewers are ignorant of the mercantile nature of the posts on Instagram [12]. Likes and comments for posts by patients on Instagram were far greater than those posted by dental practitioners. 56.2% respondents considered YouTube advertisements and channels to be a highly appreciated source of information on aligners. This follows the results of the study by Sebastian et al. (2021) and was attributed to the fact that an algorithm based on the viewer’s prior search history and frequently viewed content selected the advertisement they were recommended on Youtube [13]. YouTube provides searchable content which can help get the attention of new patients. It also helps upload instructional videos or videos of events conducted in the dental office. 54.5% respondents viewed TikTok as a considerable influencer on their decision to opt aligners.

However, the information obtained on TikTok is not necessarily reliable and of an acceptable quality as shown in the study by Meade et al. (2022) [14]. As per the results of our study, Facebook posts and pages were also common social media from where patients (50.2%) got their information on aligners because it may help provide interesting content and can be used as an effective form of inbound marketing. Facebook is easy to use and can be linked to an orthodontist’s practice website. Facebook also provides an opportunity to interact with your patients and help them stay informed about the developments in the dental office. 48.2% respondents felt that Twitter was a social media site which had a considerable influence on their decision to undergo aligner treatment. The major advantage of Twitter is accorded to its quick and easy to use content, which is limited to a 140 characters. Twitter helps orthodontists to include announcements about available appointments, practice related events and can be promoted easily on a brochure with a link to your website and other practice correspondence at the reception.

In addition, the potential risks associated with the use of social media platforms for health information and advertising should be recognised [15]. One major concern is the risk of misinformation. Social media can quickly spread non-verified or inaccurate information, thereby misleading patients about the efficacy, safety, and suitability of certain treatments, including abrasive aligners, as described by Abdelemam et al. (2024) [16]. Studies included in a systematic review by Borges et al. (2022) has pointed out that health-related misinformation is widespread on platforms such as TikTok, Instagram and YouTube, thus shaping the perception of patients and decisions that are not always consistent with evidence-based practices [17]. For example, Meade et al. (2022) found that the information on TikTok's orthodontic adjustment was often poor, with many posts lacking references and scientific support [18]. 

The increasing spread of false information poses major challenges to clinical decisions based on evidence and ethical standards in orthodontics practice [19]. Patients are increasingly turning to digital platforms for health guidance and being exposed to content that lacks scientific validation [17][18]. This often fosters unrealistic expectations about treatment outcomes, costs and duration, and can change patients' clinical suitability to aesthetics. These distortions can undermine personalized treatment planning and lead to poor results, non-compliance or dissatisfaction. Ethically, this trend threatens the principle of informed consent, as patients may be misled even before consulting professionals. Consequently, dentists must not only provide expert care but also actively participate in digital education. Through the sharing of accurate and peer-reviewed information, orthodontics can help patients make informed decisions and maintain the integrity of marketing practices.

There exist a few limitations to this study despite some of the important insights generated. Firstly, the digital nature of the survey may have excluded individuals who are less digitally literate. In addition, further study is required to have a detailed analysis on methods to achieve maximum return on investments in the case of marketing pertaining to the field of orthodontics. Furthermore, ethical concerns surrounding the rising trend of DIY aligners were not deeply explored within this study. Future research should more comprehensively address the implications and patient safety issues related to DIY orthodontic treatments.

## Conclusions

Social media have an important impact on aligned treatment decisions, particularly in women and young adults. Instagram has emerged as the most influential platform. Dental professionals should not only recognize the marketing power of social media, but also actively curate and share scientifically accurate content. Future research should focus on optimizing digital marketing strategies that balance engagement and ethical standards, as well as evaluating the safety and results of DIY aligner users affected by these platforms.

## Appendices

**Table 6:** Questionnaire in tabular format: is social media influencing patients to choose aligner treatment?

Question No.	Question	Type	Options
1	What is your age?	Single choice (oval)	- 12–19 years - 20–40 years - 40 years and above
2	Gender	Single choice (oval)	- Male - Female - Other
3	From where did you hear about aligners?	Multiple choice (tick)	- Word of Mouth - Youtube advertisements or channels - Reddit subreddits - Instagram posts, reels, stories - Tiktok - Twitter - Facebook posts and advertisements - Snapchat - Quora answers - Promotional emails - Promotional calls and SMS - OTT platform advertisements - Internet Websites - Newspaper advertisements - Magazines - Television advertisements - Billboard advertisements - Other
4	To what degree was the influence of media advertisements in you undergoing aligner treatment?	Single choice (oval)	- Very High - High - Medium - Low
5	If you have used social media for information on aligners, did you find it useful?	Single choice (oval)	- Yes - No - Maybe
6	What type of advertisement strategy do you prefer?	Multiple choice (tick)	- Word of mouth - Print advertisement - Digital advertisement
7	Do you think it’s important to scroll through relevant social media before seeking treatment?	Single choice (oval)	- Yes - No
8	Do you feel that social media made you better informed about aligners?	Single choice (oval)	- Yes - No - Maybe
